# Editorial: Neuroscientific Research for Management of Dementia

**DOI:** 10.3389/fnagi.2019.00031

**Published:** 2019-03-14

**Authors:** Mohammad A. Kamal, Fatima A. Nasrallah

**Affiliations:** ^1^King Fahd Medical Research Center, King Abdulaziz University, Jeddah, Saudi Arabia; ^2^Enzymoics, Hebersham, NSW, Australia; ^3^Novel Global Community Educational Foundation, Sydney, NSW, Australia; ^4^Queensland Brain Institute, University of Queensland, Brisbane, QLD, Australia

**Keywords:** aging, central and peripheral nervous systems, dementia, inflammation and immunology, neurodegenerative disorders, neuroscience, psychology

Each system in our body is valuable for the unique function it carries out. The nervous system for example is easily the most complex system in the human species which is sophistically made up of neurons, synapses, and various important specialized cells for appropriate signaling of neuro- transmission. Overall role of CNS is to control whole body functions in a systematic mechanism. Any abnormalities in any part of neuro-mechanism result in physiological to psychological disorder/behaviors. Therefore, researchers and medical health care staff are driven to improve physical health as well as state of psychological health in term of close connection with the nervous and immune system. The purpose of this Research Topic of Frontiers in Aging Neuroscience was to shed light on the latest outstanding discoveries pertained in wide spectrum of aging neuroscience by covering different aspects of nervous system to dementia in their original research articles and reviews. Taking this into consideration, the Research Topic on Frontiers in Neuroscientific Research for Management of Dementia by Frontiers in Aging Neuroscience makes a contribution with updates and different perspective on this important theme, developed over 17 articles (Table 1).

**Table 1:** Summaries of articles in the Research Topic on Frontiers in Neuroscientific Research for Management of Dementia.

**Authors**	**Title**	**Summary**
Cho et al.	Changes in blood factors and ultrasound findings in mild cognitive impairment and dementia	The present study aimed to assess the changes in blood factors and ultrasound measures of atherosclerosis burden patient with MCI and dementia. Peripheral blood samples and ultrasonography findings were obtained for 53 enrolled participants. Flow cytometry was used to evaluate levels of activated platelets and platelet-leukocyte aggregates (PLAs). The number of platelets expressing p-selectin was correlated with IMT (intima media thickness) and plaque number in both the MCI and dementia groups. The number of platelets expressing p-selectin glycoprotein ligand (PSGL) was strongly correlated with IMT in patients with MCI, whereas the number of platelets expressing PGSL was correlated with plaque number rather than IMT in patients with dementia. PLAs was associated with both IMT and plaque number in patients with MCI but not in those with dementia. Our findings demonstrate that alterations in IMT and plaque number are associated with an increased risk of cognitive decline as well as conversion from MCI to dementia and that blood factor analysis may aid to detect the severity of cognitive decline.
Rahman et al.	Entanglement of UPR^ER^ in Aging driven Neurodegenerative diseases	The endoplasmic reticulum (ER) is an indispensable cellular organelle that remains highly active in neuronal cells. The ER bears the load of maintaining protein homeostasis in the cellular network by managing the folding of incoming nascent peptides; however, the stress imposed by physiological/environmental factors can cause ER dysfunctions that lead to the activation of ER unfolded protein response UPR^ER^. Aging leads to deterioration of several cellular pathways and therefore weakening of the UPR^ER^. The decline in functioning ofthe UPR^ER^ during aging results in accumulation of misfolded proteins that becomes intracellular inclusions in neuronal cells, resulting in toxicity manifested as neurodegenerative diseases. With ascension in cases of neurodegenerative diseases, understanding the enigma behind aging driven UPR^ER^ dysfunction may lead to possible treatments.
Xiong et al.	Dl-3-n-butylphthalide treatment enhances hemodynamics and ameliorates memory deficits in rats with chronic cerebral hypoperfusion	Our previous study has revealed that chronic cerebral hypoperfusion (CCH) activates a compensatory vascular mechanism attempting to maintain an optimal cerebral blood flow (CBF). However, this compensation failed to prevent neuronal death and cognitive impairment because neurons die prior to the restoration of normal CBF. Therefore, pharmacological invention may be critical to enhance the CBF for reducing neurodegeneration and memory deficit. Dl-3-n-butylphthalide (NBP) is a compound isolated from the seeds of Chinese celery and has been proven to be able to prevent neuronal loss, reduce inflammation, and ameliorate memory deficits in acute ischemic animal models and stroke patients. In the present study, we used magnetic resonance imaging (MRI) techniques, immunohistochemistry, and Morris water maze to investigate whether NBP can accelerate CBF recovery, reduce neuronal death and improve cognitive deficits in chronic cerebral hypoperfusion (CCH) rats after permanent bilateral common carotid artery occlusion (BCCAO). Rats were intravenously injected with NBP (5 mg/kg) daily for 14 days beginning the first day after BCCAO. The results showed that NBP shortened recovery time of CBF to pre-occlusion levels at 2 weeks following BCCAO, compared to 4 weeks in the vehicle group, and enhanced hemodynamic compensation through dilation of the vertebral arteries and increase in angiogenesis. NBP treatment also markedly reduced reactive astrogliosis and cell apoptosis and protected hippocampal neurons against ischemic injury. The escape latency of CCH rats in the Morris water maze was also reduced in response to NBP treatment. These findings demonstrate that NBP can accelerate the recovery of CBF and improve cognitive function in a rat model of CCH, which suggesting that NBP is a promising therapy for CCH patients or vascular dementia.
Jan et al.	Perspective insights of exosomes in neurodegenerative diseases: A critical appraisal	Exosomes are small membranous entities of endocytic origin. Their production by a wide variety of cells in eukaryotes implicates their roles in the execution of essential processes, especially cellular communication. Exosomes are secreted under both physiological and pathophysiological conditions, and their actions on neighboring and distant cells lead to the modulations of cellular behaviors. They also importantly assist the delivery of disease causing entities, such as, prions, α-syn, and tau, and thus, facilitate spread to non-effected regions and accelerate the progressions of neurodegenerative diseases. The characterization of exosomes, provides information on aberrant processes, and thus, exosome analysis has many clinical applications. Because they are associated with the transport of different cellular entities across the blood-brain barrier, exosomes might be useful for delivering drugs and other therapeutic molecules to brain. Herein, we review roles played by exosomes in different neurodegenerative diseases, and the possibilities of using them as diagnostic biomarkers of disease progression, drug delivery vehicles, and in gene therapy.
Park et al.	Low serum phosphorus correlates with cerebral Aβ deposition in cognitively impaired subjects: results from the KBASE study	Alzheimer's disease (AD), characterized by progressive cognitive decline, is the most prevalent neurodegenerative disease in the elderly. Cerebral β-amyloid (Aβ) deposition is the major pathological hallmark of AD. Recent studies also have shown that the serum level of phosphorus correlates to the risk of incident dementia. To date, the linkage between cerebral Aβ deposition and the serum phosphorus level remains unknown. In this study, we analyzed the levels of serum phosphorus in 109 mild cognitive impairment (MCI) and 73 AD dementia (ADD) subjects. All subjects underwent Pittsburgh compound B positron emission tomography (PiB-PET) imaging to measure cerebral Aβ deposition. The results with Aβ deposition was compared with the serum levels of phosphorus. The subjects with cerebral Aβ deposition showed lower levels of serum phosphorus than those without Aβ deposition. Furthermore, multiple regression analyses showed that a low level of serum phosphorus correlated with cerebral Aβ deposition, even when age, sex, apolipoprotein E ε4 genotype, and MMSE z-score were controlled for. Serum levels of other ions, including calcium, iron, zinc, and copper, showed no such correlation. In conclusion, our results suggest that the serum level of phosphorus may be used as an easily accessible blood biomarker for cerebral Aβ deposition in a cognitively impaired population.
Kuang et al.	Neuroprotective effect of Ligustilide through induction of α-secretase processing of both APP and Klotho in a mouse model of Alzheimer's disease	Emerging evidence suggests that alpha-processing single transmembrane proteins, amyloid precursor protein (APP) and anti-aging protein Klotho, are likely to be involved in the progression of Alzheimer's disease (AD). The natural phthalide Ligustilide (LIG) has been demonstrated to protect against aging- and amyloid-β (Aβ)-induced brain dysfunction in animal models. The present study is to investigate the effects of LIG on cognitive deficits and metabolism of both APP and Klotho and its underlying mechanism in AD double-transgenic (APP/PS1) mice and cultured human cells. Our results show that treatment with LIG significantly ameliorated memory impairment and Aβ levels and plaques burden. Specifically, LIG might act as a potent enhancer of α-secretase, disintegrin, and metalloprotease 10 (ADAM10), leading to upregulation of alpha-processing of both APP and Klotho and subsequent increases in the levels of both soluble APP fragment (sAPPα) and soluble Klotho (sKL) with inhibition of IGF-1/Akt/mTOR signaling in AD mice and cultured cells. Moreover, the specific ADAM10 inhibitor (G1254023X) effectively reversed LIG-induced alpha-processing of both APP and Klotho *in vitro*, while Klotho gene knockdown by small interfering RNA (siRNA) significantly blunted LIG-mediated inhibition of IGF-1/Akt/mTOR signaling *in vitro*. Taken together with reported neuroprotective effects of both sAPPα and sKL as well as autophagy induction by Akt/mTOR pathway inhibition, our findings suggest that neuroprotection of LIG against AD is associated with induction alpha-processing of APP and Klotho and potential Aβ clearance. Whether LIG might induce Aβ autophagic clearance and the underlying mechanisms need to be further studied.
Yang et al.	Multiple evidences for association between cognitive impairment and dysglycemia in Parkinson's disease: implications for clinical practice	Background and purpose: It remains unclear about the etiopathogenesis of cognitive impairment (CI) in Parkinson's disease (PD). Since diabetes mellitus (DM) has been shown to be associated with CI in several diseases, we examined the association between CI and dysglycemia in PD. Methods: Enrolled PD patients completed a series of clinical and neuropsychological assessments. Motor symptoms were determined by Hohen-Yahr staging (H-Y staging) and Unified Parkinson's Disease Rating Scale—motor score (UPDRS-III). Neuropsychological functions were evaluated by the MiniMental State Examination (MMSE), the Montreal Cognitive Assessment (MoCA), and the Hamilton Anxiety and Depression Scales. Moreover, fasting glucose, fasting insulin, glycosylated hemoglobin A1c (HbA1c) and oral glucose tolerance test were performed to assess glucose metabolism. Results: MoCA and MMSE scores in PD patients with DM group (PD-DM) were significantly lower than those in PD patients without DM group (PD-nDM). Consistently, PD-DM group showed significantly higher constituent ratio of CI than PD-nDM group. In addition, MoCA scores in HbA1c ≥ 6.5% group and HbA1c ≥ 7% group were significantly lower than those in the corresponding control groups. MoCA score in IR ≥ 3 group was significantly lower than that in IR < 3 group. Furthermore, MoCA score was negatively correlated with H-Y staging, HbA1c and insulin resist ance, respectively. Finally, regression analysis indicated that H-Y staging and HbA1c ≥ 7% were independent risk factors of CI in PD. Conclusions: CI may be tightly associated with dysglycemia in, at least partially, PD patients. Importantly, H-Y staging and HbA1c ≥ 7%, two independent risk factors of CI in PD, may serve as key biomarkers in future PD clinical practice.
Islam et al.	Presence of anticardiolipin antibodies in patients with dementia: a systematic review and meta-analysis	Growing evidences are supporting toward the involvement of antiphospholipid antibodies [aPLs e.g., lupus anticoagulant (LA), anticardiolipin (aCL) and anti-β2-glycoprotein I (anti-β2-GPI) antibodies] in various neurological manifestations including migraine, epilepsy and dementia in the presence or absence of autoimmune diseases such as antiphospholipid syndrome or systemic lupus erythematosus. The aim of this systematic review and meta-analysis was to assess the presence of aPLs in dementia patients without a diagnosis of any autoimmune disease. Electronic databases (e.g., PubMed, Web of Science, Scopus, ScienceDirect, and Google Scholar) were searched without any year or language restrictions and based on the inclusion criteria, nine prospective case-control studies assessing only aCL were included involving 372 dementia patients and 337 healthy controls. No studies were found to assess the presence of both LA or anti-β2-GPI. The study-specific odds ratios (ORs) and 95% confidence intervals (CIs) were calculated using random-effects model. We observed the prevalence of aCL in dementia was higher (32.80%) than that of controls (9.50%) e.g., 3.45 times higher risk of presenting with dementia than the controls, and significant presence of aCL antibodies was detected in dementia patients compared to controls (OR: 4.94, 95% CI: 2.66–9.16, *p* < 0.00001; *I*^2^ = 32%, *p* = 0.16). Publication bias was not observed from Egger's (*p* = 0.081) and Begg's tests (*p* = 0.180). Based on the study quality assessment using modified Newcastle-Ottawa Scale for case-control studies, seven of nine studies were of high methodological quality scoring ≥ 7 (median value). In summary, aCL antibodies were significantly present in dementia patients suggesting that aCL antibodies are generated due to the autoimmune-derived effects of dementia or there might be a potential causative role of this autoantibody in dementia pathogenesis.
Moretti et al.	Vitamin D, homocysteine, and folate in subcortical vascular dementia and Alzheimer dementia	Dementia is a worldwide health problem which affects millions of patients; Alzheimer's disease (AD) and subcortical vascular dementia (sVAD) are the two most frequent forms of its presentation. As no definite therapeutic options have been discovered, different risk factors for cognitive impairment have been searched for potential therapies. This report focuses on the possible evidence that vitamin D deficiency and hyper-homocysteinemia can be considered as two important factors for the development or the progression of neurodegenerative or vascular pathologies. To this end, we assessed: the difference in vascular risk factors and vitamin D-OH25 levels among groups of sVAD, AD, and healthy age-matched controls; the association of folate, B12, homocysteine, and vitamin D with sVAD/AD and wether a deficiency of vitamin D and an increment in homocysteine levels may be related to neurodegenerative or vessel damages. The commonly-considered vascular risk factors were collected in 543 patients and compared with those obtained from a healthy old volunteer population. ANOVA group comparison showed that vitamin D deficiency was present in demented cases, as well as low levels of folate and high levels of homocysteine, more pronounced in sVAD cases. The statistical models we employed, with regression models built, and adjustments for biochemical, demographic and neuropsychiatric scores, confirmed the association between the three measures (folate decrease, hyperhomocysteinemia and vitamin D decrease) and dementia, more pronounced in sVAD than in AD.
Serino et al.	A novel Virtual Reality-based training protocol for the enhancement of the “mental frame syncing” in individuals with Alzheimer's Disease: a development-of-concept trial	A growing body of evidence suggests that people with Alzheimer's Disease (AD) show compromised spatial abilities. In addition, there exists from the earliest stages of AD a specific impairment in “mental frame syncing,” which is the ability to synchronize an allocentric viewpoint-independent representation (including object-to-object information) with an egocentric one by computing the bearing of each relevant “object” in the environment in relation to the stored heading in space (i.e., information about our viewpoint contained in the allocentric viewpoint-dependent representation). The main objective of this development-of-concept trial was to evaluate the efficacy of a novel VR-based training protocol focused on the enhancement of the “mental frame syncing” of the different spatial representations in subjects with AD. We recruited 20 individuals with AD who were randomly assigned to either “VR-based training” or “Control Group.” Moreover, eight cognitively healthy elderly individuals were recruited to participate in the VR-based training in order to have a different comparison group. Based on a neuropsychological assessment, our results indicated a significant improvement in long-term spatial memory after the VR-based training for patients with AD; this means that transference of improvements from the VR-based training to more general aspects of spatial cognition was observed. Interestingly, there was also a significant effect of VR-based training on executive functioning for cognitively healthy elderly individuals. In sum, VR could be considered as an advanced embodied tool suitable for treating spatial recall impairments.
Gong et al.	Chronic monoarthritis pain accelerates the processes of cognitive impairment and increases the NMDAR subunits NR2B in CA3 of hippocampus from 5-month-old transgenic APP/PS1 mice	Many factors impact cognitive impairment; however, the effects of chronic pain and the mechanisms underlying these effects on cognitive impairment are currently unknown. Here we tested the hypothesis that chronic pain accelerates the transition from normal cognition to mild cognitive impairment in 5-month-old transgenic APP/PS1 mice, an animal model of Alzheimer's disease, and that neurotoxicity induced by N-methyl-D-aspartic acid receptor (NMDAR) subunits may be involved in this process. Chronic monoarthritis pain was induced in transgenic APP/PS1 mice and 5-month-old wild-type mice by intra- and pre-articular injections of Freund's complete adjuvant into one knee joint. Pain behavior, learning and memory function, and the distribution and quantity of NMDAR subunits (NR1, NR2A and NR2B) in hippocampal CA1 and CA3 regions were assessed. Our results showed that although persistent and robust monoarthritis pain was induced by the Freund's complete adjuvant injections, only the transgenic APP/PS1 mice with chronic monoarthritis pain exhibited marked learning and memory impairments. This result suggested that chronic monoarthritis pain accelerated the cognitive impairment process. Furthermore, only transgenic APP/PS1 mice with chronic monoarthritis pain exhibited an overexpression of NR2B and an increased NR2B/NR2A ratio in the hippocampus CA3. These findings suggest that chronic pain is a risk factor for cognitive impairment and that increased neurotoxicity associated with NMDAR subunit activation may underpin the impairment. Thus, NMDARs may be a therapeutic target for the prevention of chronic pain-induced cognitive impairment.
Alexiou et al.	A Bayesian model for the prediction and early diagnosis of Alzheimer's disease	Alzheimer's disease treatment is still an open problem. The diversity of symptoms, the alterations in common pathophysiology, the existence of asymptomatic cases, the different types of sporadic and familial Alzheimer's and their relevance with other types of dementia and comorbidities, have already created a myth-fear against the leading disease of the twenty-first century. Many failed latest clinical trials and novel medications have revealed the early diagnosis as the most critical treatment solution, even though scientists tested the amyloid hypothesis and few related drugs. Unfortunately, latest studies have indicated that the disease begins at the very young ages thus making it difficult to determine the right time of proper treatment. By taking into consideration all these multivariate aspects and unreliable factors against an appropriate treatment, we focused our research on a non-classic statistical evaluation of the most known and accepted Alzheimer's biomarkers. Therefore, in this paper, the code and few experimental results of a computational Bayesian tool have been reported, dedicated to the correlation and assessment of several Alzheimer's biomarkers to export a probabilistic medical prognostic process. This new statistical software is executable in the Bayesian software Winbugs, based on the latest Alzheimer's classification and the formulation of the known relative probabilities of the various biomarkers, correlated with Alzheimer's progression, through a set of discrete distributions. A user-friendly web page has been implemented for the supporting of medical doctors and researchers, to upload Alzheimer's tests and receive statistics on the occurrence of Alzheimer's disease development or presence, due to abnormal testing in one or more biomarkers.
Vieira et al.	Protein Tyrosine Phosphatase 1B (PTP1B): A Potential Target for Alzheimer's Therapy?	Despite significant advances in current understanding of mechanisms of pathogenesis in Alzheimer's disease (AD), attempts at drug development based on those discoveries have failed to translate into effective, disease-modifying therapies. AD is a complex and multifactorial disease comprising a range of aberrant cellular/molecular processes taking part in different cell types and brain regions. As a consequence, therapeutics for AD should be able to block or compensate multiple abnormal pathological events. Here, we examine recent evidence that inhibition of protein tyrosine phosphatase 1B (PTP1B) may represent a promising strategy to combat a variety of AD-related detrimental processes. Besides its well described role as a negative regulator of insulin and leptin signaling, PTB1B recently emerged as a modulator of various other processes in the central nervous system (CNS) that are also implicated in AD. These include signaling pathways germane to learning and memory, regulation of synapse dynamics, endoplasmic reticulum stress and microglia-mediated neuroinflammation. We propose that PTP1B inhibition may represent an attractive and yet unexplored therapeutic approach to correct aberrant signaling pathways linked to AD.
Zheng et al.	Plasma Exosomes Spread and Cluster Around β-Amyloid Plaques in an Animal Model of Alzheimer's Disease	Exosomes, a type of extracellular vesicle, have been shown to be involved in many disorders, including Alzheimer's disease. Exosomes may contribute to the spread of misfolded proteins such as amyloid-β and α-synuclein. However, the specific diffusion process of exosomes and their final destination in brain are still unclear. In the present study, we isolated exosomes from peripheral plasma and injected them into the hippocampus of an Alzheimer's disease mouse model, and investigated exosome diffusion. We found that injected exosomes can spread from the dentate gyrus to other regions of hippocampus and to the cortex. Exosomes targeted microglia preferentially; this phenomenon is stable and is not affected by age. In Alzheimer's disease mice, microglia take up lower levels of exosomes. More interestingly, plasma exosomes cluster around the amyloid-β plaques and are engulfed by activated microglia nearby. Our data indicate that exosomes can diffuse throughout the brain and may play a role in the dynamics of amyloid deposition in Alzheimer's disease through microglia.
Xu et al.	The Impact of Microbiota-Gut-Brain Axis on Diabetic Cognition Impairment	Progressive cognitive dysfunction is a central characteristic of diabetic encephalopathy (DE). With an aging population, the incidence of DE is rising and it has become a major threat that seriously affects public health. Studies within this decade have indicated the important role of risk factors such as oxidative stress and inflammation on the development of cognitive impairment. With the recognition of the two-way communication between gut and brain, recent investigation suggests that “microbiota-gut-brain axis” also plays a pivotal role in modulating both cognition function and endocrine stability. This review aims to systemically elucidate the underlying impact of diabetes on cognitive impairment.
Wang et al.	YXQN Reduces Alzheimer's Disease-like Pathology and Cognitive Decline in APPswePS1dE9 Transgenic Mice	Alzheimer's disease (AD) is the world's most common form of dementia, in which aggregation of amyloid-β (Aβ) is the hallmark. Unfortunately, few medicines have succeeded to completely cure AD. Yangxue Qingnao (YXQN) is a Chinese traditional medicine, and its pharmacological effect is improving cerebral blood flow. In this study, we firstly demonstrated that YXQN reduced AD-like pathology and cognitive impairment in APPswePS1dE9 (APP/PS1) mice with two months administration. Our data showed that YXQN substantially ameliorated behavioral defects in 10-month old APP/PS1 mice using Morris Water Maze and Y-maze tests, in which the cognitive ability of YXQN high-dose group approaches to wild type mice. Next, we focused on the brain pathological alterations in the YXQN group by three experiments, including thioflavin-S, congo-red, and Aβ-immunohistochemistry staining. The results demonstrated that the high-dose of YXQN dramatically suppressed amyloid plaques in the hippocampus and cortex of APP/PS1 mice, which showed a 47-72% reduction in plaque deposits, relative to the vehicle group. In addition, our data verified that YXQN decreased the cerebral amyloid load by attenuating β-secretase BACE1 and γ-secretase PS1 in the pathological processing of APP, and promoting the level of α-secretase ADAM10 in the physiological processing of APP to generate more sAPPα, which combats amyloidosis formation, and also carries out neurotropic and neuroprotective effect. Taken together, our results strongly suggest that YXQN could be a potential medicine for AD, and provide new evidence for further AD drug research and development.
Cozac et al.	Increase of EEG spectral theta power indicates higher risk of the development of severe cognitive decline in Parkinson's disease after 3 years	Objective: We investigated quantitative electroencephalography (qEEG) and clinical parameters as potential risk factors of severe cognitive decline in Parkinson's disease. Methods: We prospectively investigated 37 patients with Parkinson's disease at baseline and follow-up (after 3 years). Patients had no severe cognitive impairment at baseline. We used a summary score of cognitive tests as the outcome at follow-up. At baseline we assessed motor, cognitive, and psychiatric factors; qEEG variables (global relative median power spectra) were obtained by a fully automated processing of high-resolution EEG (256-channels). We used linear regression models with calculation of the explained variance to evaluate the relation of baseline parameters with cognitive deterioration. Results: The following baseline parameters significantly predicted severe cognitive decline: global relative median power theta (4–8 Hz), cognitive task performance in executive functions and working memory. Conclusions: Combination of neurocognitive tests and qEEG improves identification of patients with higher risk of cognitive decline in PD.

We hope that this Frontiers Research Topic will be an enrichment for Neuroscientific Research for Management of Dementia, with the efforts and commitment of all authors to whom we give our acknowledgment as well as to the reviewers who have contributed in improving and clarifying these diverse contributions due to their valuable comments. Finally, a special thanks to Editor in Chief and Frontiers management team for support in publishing process.

## Author Contributions

All authors listed have made a substantial, direct and intellectual contribution to the work, and approved it for publication.

### Conflict of Interest Statement

The authors declare that the research was conducted in the absence of any commercial or financial relationships that could be construed as a potential conflict of interest.

